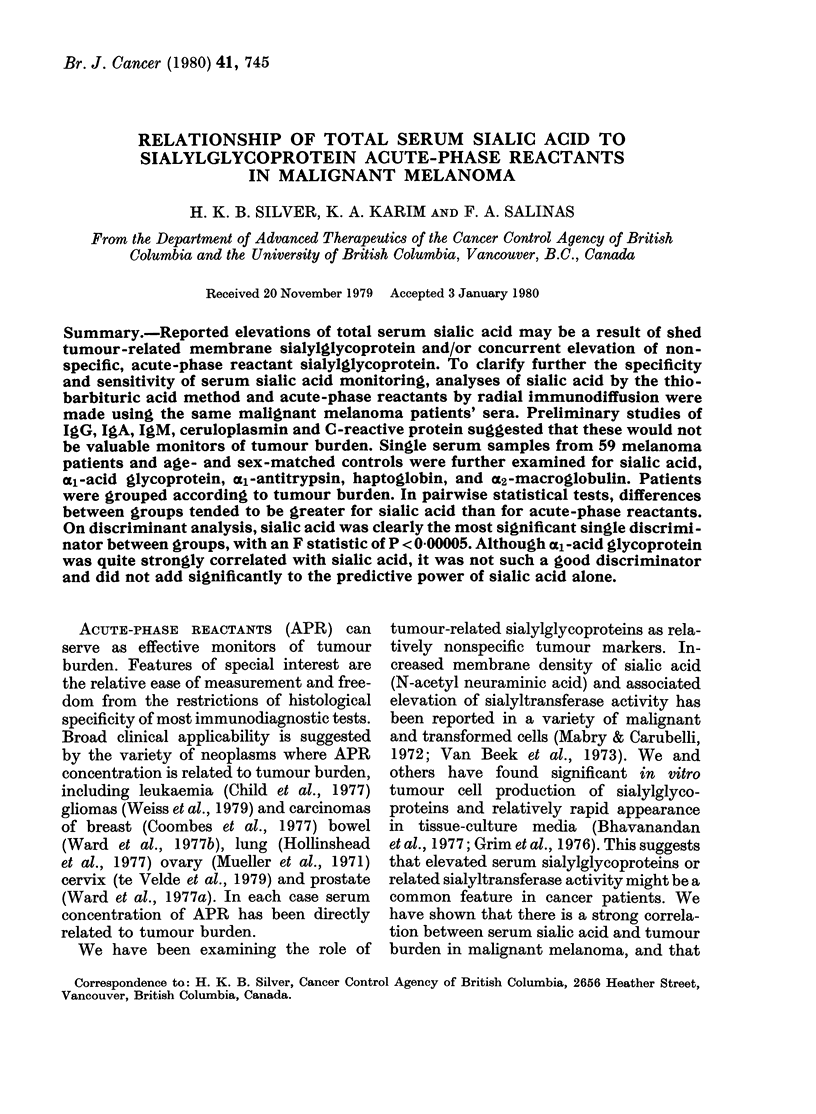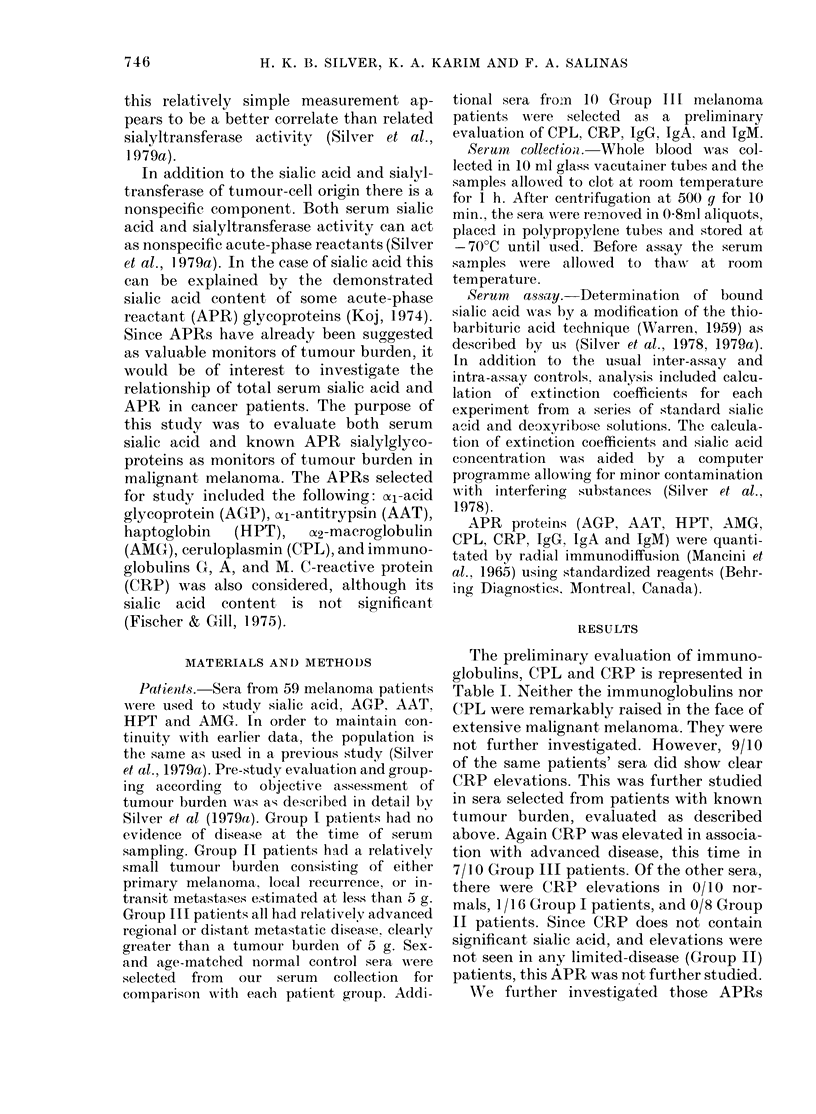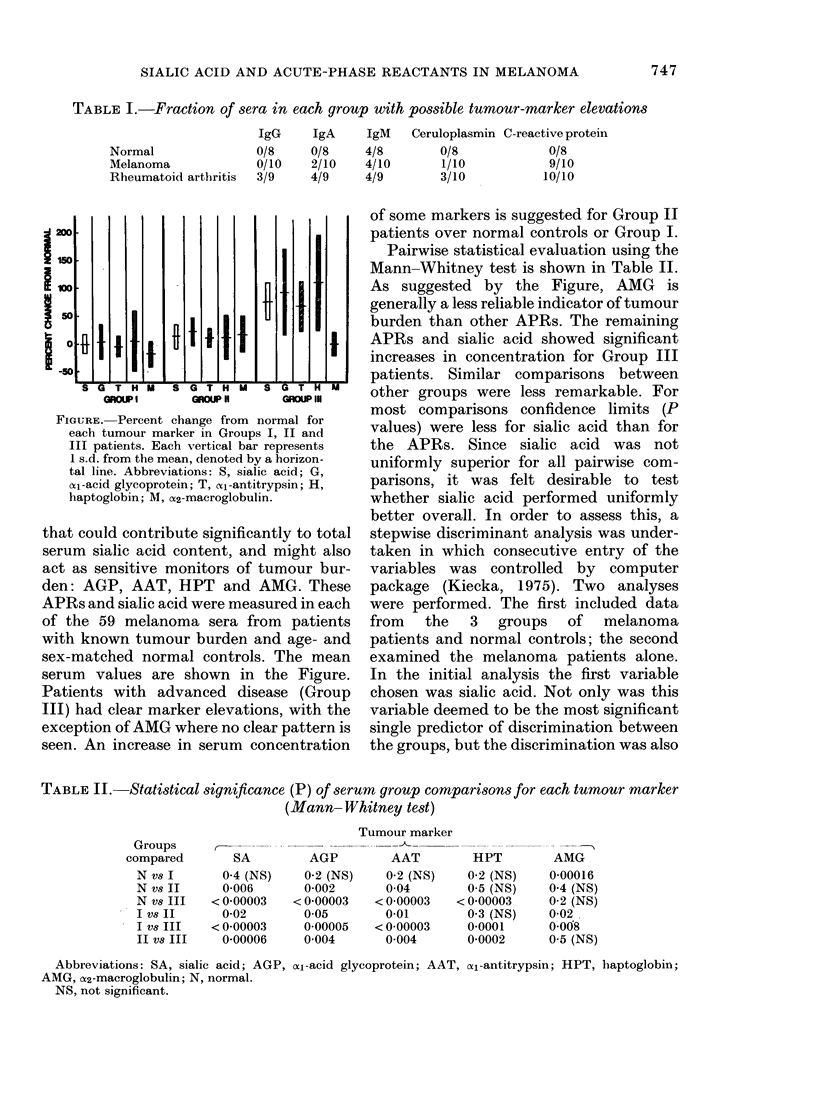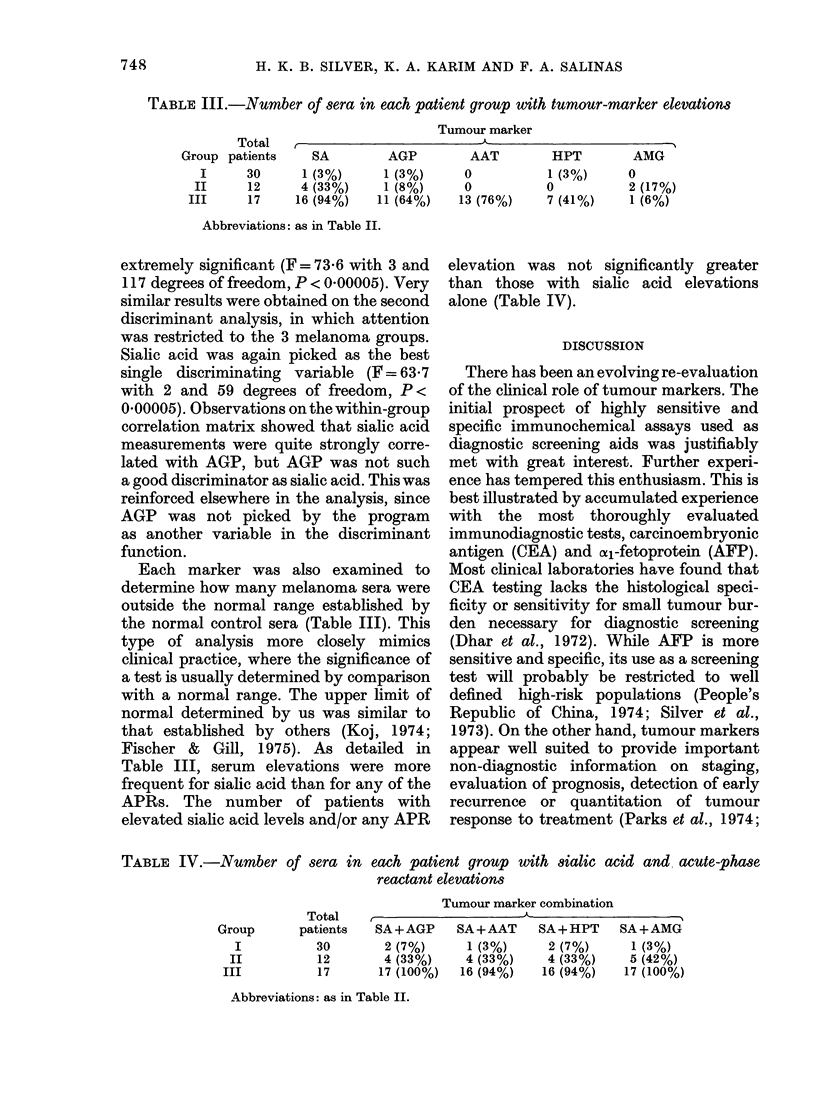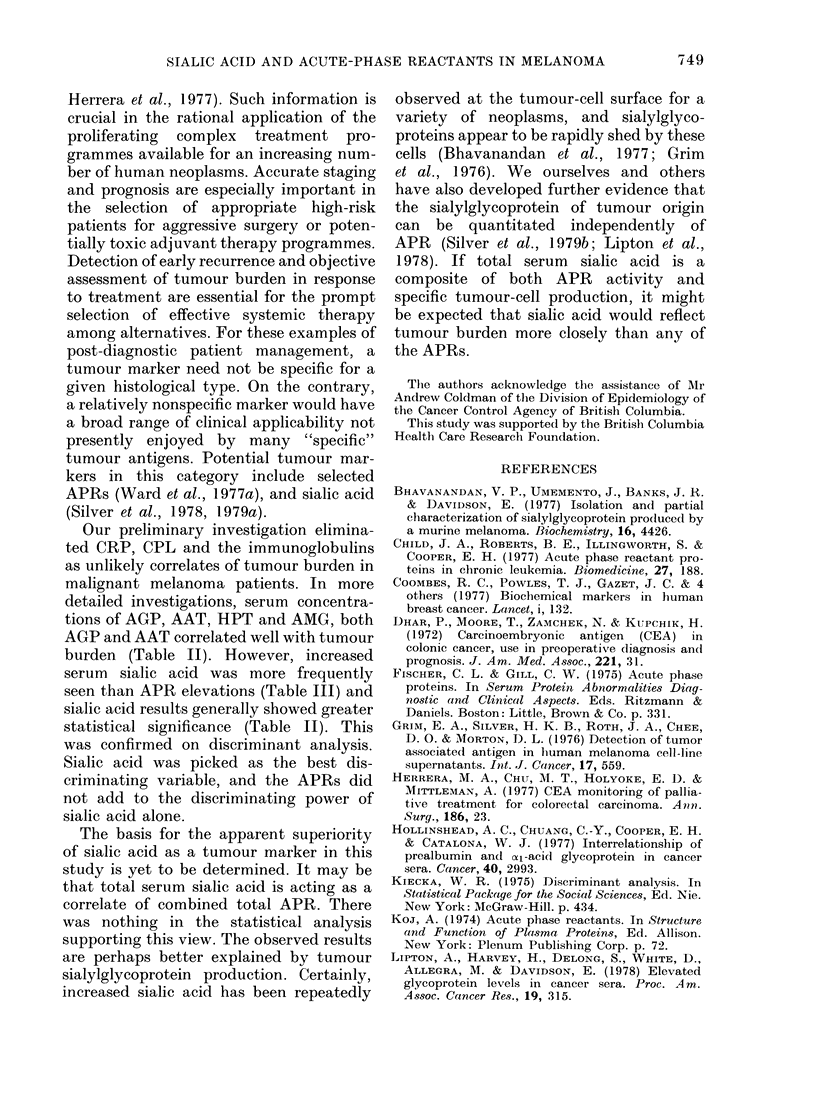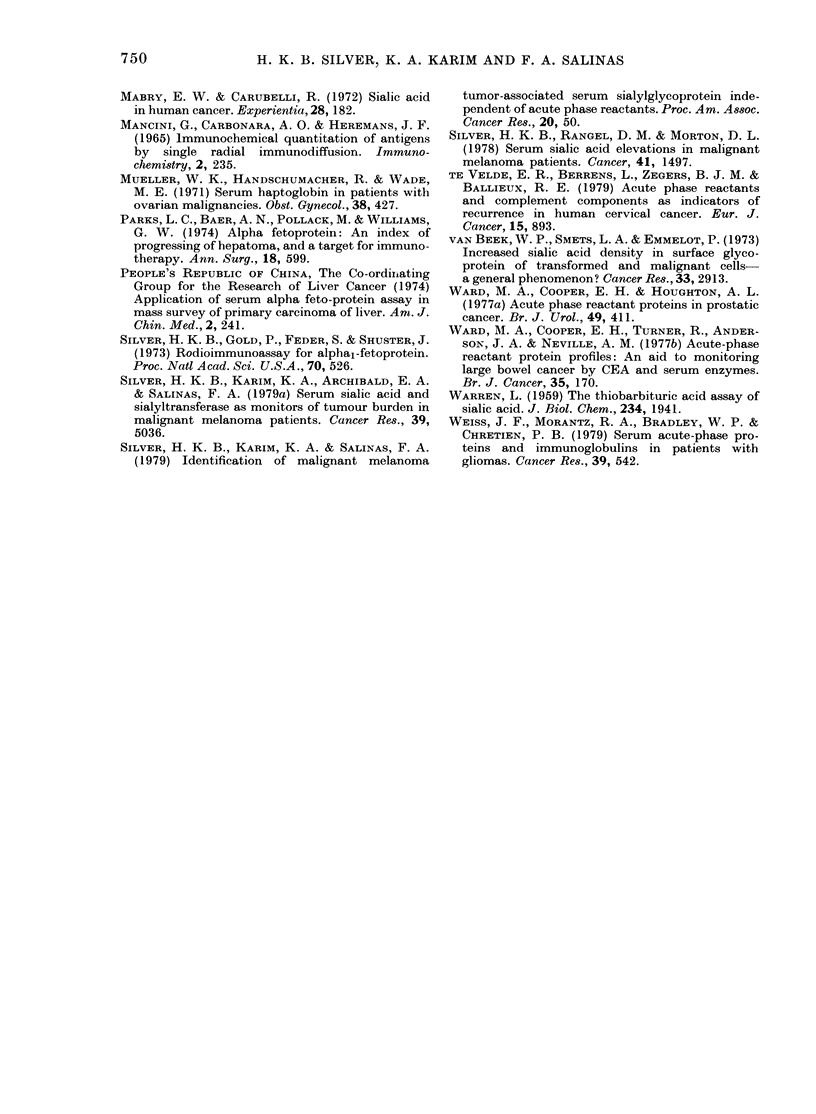# Relationship of total serum sialic acid to sialylglycoprotein acute-phase reactants in malignant melanoma.

**DOI:** 10.1038/bjc.1980.136

**Published:** 1980-05

**Authors:** H. K. Silver, K. A. Karim, F. A. Salinas

## Abstract

Reported elevations of total serum sialic acid may be a result of shed tumour-related membrane sialyglycoprotein and/or concurrent elevation of non-specific, acute-phase reactant sialoglycoprotein. To clarify further the specificity and sensitivity of serum sialic acid monitoring, analyses of sialic acid by the thiobarbituric acid method and acute-phase reactants by radial immunodiffusion were made using the same malignant melanoma patients' sera. Preliminary studies of IgG, IgA, IgM, ceruloplasmin and C-reactive protein suggested that these would not be valuable monitors of tumour burden. Single serum samples from 59 melanoma patients and age- and sex-matched controls were further examined for sialic acid, alpha 1-acid glycoprotein, alpha 1-antitrypsin, haptoglobin, and alpha 2-macroglobulin. Patients were grouped according to tumour burden. In pairwise statistical tests, differences between groups tended to be greater for sialic acid than for acute-phase reactants. On discriminant analysis , sialic acid was clearly the most significant single discriminator between groups, with an F statistic of P < 0.00005. Although alpha 1-acid glycoprotein was quite strongly correlated with sialic acid, it was not such a good discriminator and did not add significantly to the predictive power of sialic acid alone.


*Br. J. Cancer* (1980) **41**, 745

### RELATIONSHIP OF TOTAL SERUM SIALIC ACID TO SIALYLGLYCOPROTEIN ACUTE-PHASE REACTANTS IN MALIGNANT MELANOMA

H. K. B. SILVER, K. A. KARIM AND F. A. SALINAS

*From the Department of Advanced Therapeutics of the Cancer Control Agency of British Columbia and the University of British Columbia, Vancouver, B.C., Canada*

Received 20 November 1979 Accepted 3 January 1980

**Summary.—Reported elevations of total serum sialic acid may be a result of shed tumour-related membrane sialylglycoprotein and/or concurrent elevation of non-specific, acute-phase reactant sialylglycoprotein. To clarify further the specificity and sensitivity of serum sialic acid monitoring, analyses of sialic acid by the thiobarbituric acid method and acute-phase reactants by radial immunodiffusion were made using the same malignant melanoma patients' sera. Preliminary studies of IgG, IgA, IgM, ceruloplasmin and C-reactive protein suggested that these would not be valuable monitors of tumour burden. Single serum samples from 59 melanoma patients and age- and sex-matched controls were further examined for sialic acid, $\alpha_1$-acid glycoprotein, $\alpha_1$-antitrypsin, haptoglobin, and $\alpha_2$-macroglobulin. Patients were grouped according to tumour burden. In pairwise statistical tests, differences between groups tended to be greater for sialic acid than for acute-phase reactants. On discriminant analysis, sialic acid was clearly the most significant single discriminator between groups, with an F statistic of $P < 0{\cdot}00005$. Although $\alpha_1$-acid glycoprotein was quite strongly correlated with sialic acid, it was not such a good discriminator and did not add significantly to the predictive power of sialic acid alone.**

ACUTE-PHASE REACTANTS (APR) can serve as effective monitors of tumour burden. Features of special interest are the relative ease of measurement and freedom from the restrictions of histological specificity of most immunodiagnostic tests. Broad clinical applicability is suggested by the variety of neoplasms where APR concentration is related to tumour burden, including leukaemia (Child *et al.*, 1977) gliomas (Weiss *et al.*, 1979) and carcinomas of breast (Coombes *et al.*, 1977) bowel (Ward *et al.*, 1977*b*), lung (Hollinshead *et al.*, 1977) ovary (Mueller *et al.*, 1971) cervix (te Velde *et al.*, 1979) and prostate (Ward *et al.*, 1977*a*). In each case serum concentration of APR has been directly related to tumour burden.

We have been examining the role of tumour-related sialylglycoproteins as relatively nonspecific tumour markers. Increased membrane density of sialic acid (N-acetyl neuraminic acid) and associated elevation of sialyltransferase activity has been reported in a variety of malignant and transformed cells (Mabry & Carubelli, 1972; Van Beek *et al.*, 1973). We and others have found significant *in vitro* tumour cell production of sialylglycoproteins and relatively rapid appearance in tissue-culture media (Bhavanandan *et al.*, 1977; Grim *et al.*, 1976). This suggests that elevated serum sialylglycoproteins or related sialyltransferase activity might be a common feature in cancer patients. We have shown that there is a strong correlation between serum sialic acid and tumour burden in malignant melanoma, and that

Correspondence to: H. K. B. Silver, Cancer Control Agency of British Columbia, 2656 Heather Street, Vancouver, British Columbia, Canada.

this relatively simple measurement appears to be a better correlate than related sialyltransferase activity (Silver *et al.*, 1979*a*).

In addition to the sialic acid and sialyltransferase of tumour-cell origin there is a nonspecific component. Both serum sialic acid and sialyltransferase activity can act as nonspecific acute-phase reactants (Silver *et al.*, 1979*a*). In the case of sialic acid this can be explained by the demonstrated sialic acid content of some acute-phase reactant (APR) glycoproteins (Koj, 1974). Since APRs have already been suggested as valuable monitors of tumour burden, it would be of interest to investigate the relationship of total serum sialic acid and APR in cancer patients. The purpose of this study was to evaluate both serum sialic acid and known APR sialylglycoproteins as monitors of tumour burden in malignant melanoma. The APRs selected for study included the following: $\alpha_1$-acid glycoprotein (AGP), $\alpha_1$-antitrypsin (AAT), haptoglobin (HPT), $\alpha_2$-macroglobulin (AMG), ceruloplasmin (CPL), and immunoglobulins G, A, and M. C-reactive protein (CRP) was also considered, although its sialic acid content is not significant (Fischer & Gill, 1975).

### MATERIALS AND METHODS

*Patients.*—Sera from 59 melanoma patients were used to study sialic acid, AGP, AAT, HPT and AMG. In order to maintain continuity with earlier data, the population is the same as used in a previous study (Silver *et al.*, 1979*a*). Pre-study evaluation and grouping according to objective assessment of tumour burden was as described in detail by Silver *et al* (1979*a*). Group I patients had no evidence of disease at the time of serum sampling. Group II patients had a relatively small tumour burden consisting of either primary melanoma, local recurrence, or in-transit metastases estimated at less than 5 g. Group III patients all had relatively advanced regional or distant metastatic disease, clearly greater than a tumour burden of 5 g. Sex- and age-matched normal control sera were selected from our serum collection for comparison with each patient group. Additional sera from 10 Group III melanoma patients were selected as a preliminary evaluation of CPL, CRP, IgG, IgA, and IgM.

*Serum collection.*—Whole blood was collected in 10 ml glass vacutainer tubes and the samples allowed to clot at room temperature for 1 h. After centrifugation at 500 *g* for 10 min., the sera were removed in 0·8ml aliquots, placed in polypropylene tubes and stored at −70°C until used. Before assay the serum samples were allowed to thaw at room temperature.

*Serum assay.*—Determination of bound sialic acid was by a modification of the thiobarbituric acid technique (Warren, 1959) as described by us (Silver *et al.*, 1978, 1979*a*). In addition to the usual inter-assay and intra-assay controls, analysis included calculation of extinction coefficients for each experiment from a series of standard sialic acid and deoxyribose solutions. The calculation of extinction coefficients and sialic acid concentration was aided by a computer programme allowing for minor contamination with interfering substances (Silver *et al.*, 1978).

APR proteins (AGP, AAT, HPT, AMG, CPL, CRP, IgG, IgA and IgM) were quantitated by radial immunodiffusion (Mancini *et al.*, 1965) using standardized reagents (Behring Diagnostics, Montreal, Canada).

### RESULTS

The preliminary evaluation of immunoglobulins, CPL and CRP is represented in Table I. Neither the immunoglobulins nor CPL were remarkably raised in the face of extensive malignant melanoma. They were not further investigated. However, 9/10 of the same patients' sera did show clear CRP elevations. This was further studied in sera selected from patients with known tumour burden, evaluated as described above. Again CRP was elevated in association with advanced disease, this time in 7/10 Group III patients. Of the other sera, there were CRP elevations in 0/10 normals, 1/16 Group I patients, and 0/8 Group II patients. Since CRP does not contain significant sialic acid, and elevations were not seen in any limited-disease (Group II) patients, this APR was not further studied.

We further investigated those APRs

TABLE I.—*Fraction of sera in each group with possible tumour-marker elevations*

| | IgG | IgA | IgM | Ceruloplasmin | C-reactive protein |
|---|---|---|---|---|---|
| Normal | 0/8 | 0/8 | 4/8 | 0/8 | 0/8 |
| Melanoma | 0/10 | 2/10 | 4/10 | 1/10 | 9/10 |
| Rheumatoid arthritis | 3/9 | 4/9 | 4/9 | 3/10 | 10/10 |

FIGURE.—Percent change from normal for each tumour marker in Groups I, II and III patients. Each vertical bar represents 1 s.d. from the mean, denoted by a horizontal line. Abbreviations: S, sialic acid; G, $\alpha_1$-acid glycoprotein; T, $\alpha_1$-antitrypsin; H, haptoglobin; M, $\alpha_2$-macroglobulin.

that could contribute significantly to total serum sialic acid content, and might also act as sensitive monitors of tumour burden: AGP, AAT, HPT and AMG. These APRs and sialic acid were measured in each of the 59 melanoma sera from patients with known tumour burden and age- and sex-matched normal controls. The mean serum values are shown in the Figure. Patients with advanced disease (Group III) had clear marker elevations, with the exception of AMG where no clear pattern is seen. An increase in serum concentration of some markers is suggested for Group II patients over normal controls or Group I.

Pairwise statistical evaluation using the Mann–Whitney test is shown in Table II. As suggested by the Figure, AMG is generally a less reliable indicator of tumour burden than other APRs. The remaining APRs and sialic acid showed significant increases in concentration for Group III patients. Similar comparisons between other groups were less remarkable. For most comparisons confidence limits (*P* values) were less for sialic acid than for the APRs. Since sialic acid was not uniformly superior for all pairwise comparisons, it was felt desirable to test whether sialic acid performed uniformly better overall. In order to assess this, a stepwise discriminant analysis was undertaken in which consecutive entry of the variables was controlled by computer package (Kiecka, 1975). Two analyses were performed. The first included data from the 3 groups of melanoma patients and normal controls; the second examined the melanoma patients alone. In the initial analysis the first variable chosen was sialic acid. Not only was this variable deemed to be the most significant single predictor of discrimination between the groups, but the discrimination was also

TABLE II.—*Statistical significance* (P) *of serum group comparisons for each tumour marker* (*Mann–Whitney test*)

| Groups compared | Tumour marker: SA | AGP | AAT | HPT | AMG |
|---|---|---|---|---|---|
| N *vs* I | 0·4 (NS) | 0·2 (NS) | 0·2 (NS) | 0·2 (NS) | 0·00016 |
| N *vs* II | 0·006 | 0·002 | 0·04 | 0·5 (NS) | 0·4 (NS) |
| N *vs* III | < 0·00003 | < 0·00003 | < 0·00003 | < 0·00003 | 0·2 (NS) |
| I *vs* II | 0·02 | 0·05 | 0·01 | 0·3 (NS) | 0·02 |
| I *vs* III | < 0·00003 | 0·00005 | < 0·00003 | 0·0001 | 0·008 |
| II *vs* III | 0·00006 | 0·004 | 0·004 | 0·0002 | 0·5 (NS) |

Abbreviations: SA, sialic acid; AGP, $\alpha_1$-acid glycoprotein; AAT, $\alpha_1$-antitrypsin; HPT, haptoglobin; AMG, $\alpha_2$-macroglobulin; N, normal.

NS, not significant.

TABLE III.—*Number of sera in each patient group with tumour-marker elevations*

| Group | Total patients | Tumour marker SA | AGP | AAT | HPT | AMG |
|---|---|---|---|---|---|---|
| I | 30 | 1 (3%) | 1 (3%) | 0 | 1 (3%) | 0 |
| II | 12 | 4 (33%) | 1 (8%) | 0 | 0 | 2 (17%) |
| III | 17 | 16 (94%) | 11 (64%) | 13 (76%) | 7 (41%) | 1 (6%) |

Abbreviations: as in Table II.

extremely significant (F = 73·6 with 3 and 117 degrees of freedom, $P < 0{\cdot}00005$). Very similar results were obtained on the second discriminant analysis, in which attention was restricted to the 3 melanoma groups. Sialic acid was again picked as the best single discriminating variable (F = 63·7 with 2 and 59 degrees of freedom, $P < 0{\cdot}00005$). Observations on the within-group correlation matrix showed that sialic acid measurements were quite strongly correlated with AGP, but AGP was not such a good discriminator as sialic acid. This was reinforced elsewhere in the analysis, since AGP was not picked by the program as another variable in the discriminant function.

Each marker was also examined to determine how many melanoma sera were outside the normal range established by the normal control sera (Table III). This type of analysis more closely mimics clinical practice, where the significance of a test is usually determined by comparison with a normal range. The upper limit of normal determined by us was similar to that established by others (Koj, 1974; Fischer & Gill, 1975). As detailed in Table III, serum elevations were more frequent for sialic acid than for any of the APRs. The number of patients with elevated sialic acid levels and/or any APR elevation was not significantly greater than those with sialic acid elevations alone (Table IV).

### DISCUSSION

There has been an evolving re-evaluation of the clinical role of tumour markers. The initial prospect of highly sensitive and specific immunochemical assays used as diagnostic screening aids was justifiably met with great interest. Further experience has tempered this enthusiasm. This is best illustrated by accumulated experience with the most thoroughly evaluated immunodiagnostic tests, carcinoembryonic antigen (CEA) and $\alpha_1$-fetoprotein (AFP). Most clinical laboratories have found that CEA testing lacks the histological specificity or sensitivity for small tumour burden necessary for diagnostic screening (Dhar *et al.*, 1972). While AFP is more sensitive and specific, its use as a screening test will probably be restricted to well defined high-risk populations (People's Republic of China, 1974; Silver *et al.*, 1973). On the other hand, tumour markers appear well suited to provide important non-diagnostic information on staging, evaluation of prognosis, detection of early recurrence or quantitation of tumour response to treatment (Parks *et al.*, 1974;

TABLE IV.—*Number of sera in each patient group with sialic acid and acute-phase reactant elevations*

| Group | Total patients | Tumour marker combination SA+AGP | SA+AAT | SA+HPT | SA+AMG |
|---|---|---|---|---|---|
| I | 30 | 2 (7%) | 1 (3%) | 2 (7%) | 1 (3%) |
| II | 12 | 4 (33%) | 4 (33%) | 4 (33%) | 5 (42%) |
| III | 17 | 17 (100%) | 16 (94%) | 16 (94%) | 17 (100%) |

Abbreviations: as in Table II.

Herrera *et al.*, 1977). Such information is crucial in the rational application of the proliferating complex treatment programmes available for an increasing number of human neoplasms. Accurate staging and prognosis are especially important in the selection of appropriate high-risk patients for aggressive surgery or potentially toxic adjuvant therapy programmes. Detection of early recurrence and objective assessment of tumour burden in response to treatment are essential for the prompt selection of effective systemic therapy among alternatives. For these examples of post-diagnostic patient management, a tumour marker need not be specific for a given histological type. On the contrary, a relatively nonspecific marker would have a broad range of clinical applicability not presently enjoyed by many "specific" tumour antigens. Potential tumour markers in this category include selected APRs (Ward *et al.*, 1977*a*), and sialic acid (Silver *et al.*, 1978, 1979*a*).

Our preliminary investigation eliminated CRP, CPL and the immunoglobulins as unlikely correlates of tumour burden in malignant melanoma patients. In more detailed investigations, serum concentrations of AGP, AAT, HPT and AMG, both AGP and AAT correlated well with tumour burden (Table II). However, increased serum sialic acid was more frequently seen than APR elevations (Table III) and sialic acid results generally showed greater statistical significance (Table II). This was confirmed on discriminant analysis. Sialic acid was picked as the best discriminating variable, and the APRs did not add to the discriminating power of sialic acid alone.

The basis for the apparent superiority of sialic acid as a tumour marker in this study is yet to be determined. It may be that total serum sialic acid is acting as a correlate of combined total APR. There was nothing in the statistical analysis supporting this view. The observed results are perhaps better explained by tumour sialylglycoprotein production. Certainly, increased sialic acid has been repeatedly observed at the tumour-cell surface for a variety of neoplasms, and sialylglycoproteins appear to be rapidly shed by these cells (Bhavanandan *et al.*, 1977; Grim *et al.*, 1976). We ourselves and others have also developed further evidence that the sialylglycoprotein of tumour origin can be quantitated independently of APR (Silver *et al.*, 1979*b*; Lipton *et al.*, 1978). If total serum sialic acid is a composite of both APR activity and specific tumour-cell production, it might be expected that sialic acid would reflect tumour burden more closely than any of the APRs.

The authors acknowledge the assistance of Mr Andrew Coldman of the Division of Epidemiology of the Cancer Control Agency of British Columbia.

This study was supported by the British Columbia Health Care Research Foundation.

### REFERENCES

Bhavanandan, V. P., Umemento, J., Banks, J. R. & Davidson, E. (1977) Isolation and partial characterization of sialylglycoprotein produced by a murine melanoma. *Biochemistry*, **16**, 4426.

Child, J. A., Roberts, B. E., Illingworth, S. & Cooper, E. H. (1977) Acute phase reactant proteins in chronic leukemia. *Biomedicine*, **27**, 188.

Coombes, R. C., Powles, T. J., Gazet, J. C. & 4 others (1977) Biochemical markers in human breast cancer. *Lancet*, i, 132.

Dhar, P., Moore, T., Zamchek, N. & Kupchik, H. (1972) Carcinoembryonic antigen (CEA) in colonic cancer, use in preoperative diagnosis and prognosis. *J. Am. Med. Assoc.*, **221**, 31.

Fischer, C. L. & Gill, C. W. (1975) Acute phase proteins. In *Serum Protein Abnormalities Diagnostic and Clinical Aspects*. Eds. Ritzmann & Daniels. Boston: Little, Brown & Co. p. 331.

Grim, E. A., Silver, H. K. B., Roth, J. A., Chee, D. O. & Morton, D. L. (1976) Detection of tumor associated antigen in human melanoma cell-line supernatants. *Int. J. Cancer*, **17**, 559.

Herrera, M. A., Chu, M. T., Holyoke, E. D. & Mittleman, A. (1977) CEA monitoring of palliative treatment for colorectal carcinoma. *Ann. Surg.*, **186**, 23.

Hollinshead, A. C., Chuang, C.-Y., Cooper, E. H. & Catalona, W. J. (1977) Interrelationship of prealbumin and $\alpha_1$-acid glycoprotein in cancer sera. *Cancer*, **40**, 2993.

Kiecka, W. R. (1975) Discriminant analysis. In *Statistical Package for the Social Sciences*, Ed. Nie. New York: McGraw-Hill. p. 434.

Koj, A. (1974) Acute phase reactants. In *Structure and Function of Plasma Proteins*, Ed. Allison. New York: Plenum Publishing Corp. p. 72.

Lipton, A., Harvey, H., Delong, S., White, D., Allegra, M. & Davidson, E. (1978) Elevated glycoprotein levels in cancer sera. *Proc. Am. Assoc. Cancer Res.*, **19**, 315.

MABRY, E. W. & CARUBELLI, R. (1972) Sialic acid in human cancer. *Experientia*, **28**, 182.

MANCINI, G., CARBONARA, A. O. & HEREMANS, J. F. (1965) Immunochemical quantitation of antigens by single radial immunodiffusion. *Immunochemistry*, **2**, 235.

MUELLER, W. K., HANDSCHUMACHER, R. & WADE, M. E. (1971) Serum haptoglobin in patients with ovarian malignancies. *Obst. Gynecol.*, **38**, 427.

PARKS, L. C., BAER, A. N., POLLACK, M. & WILLIAMS, G. W. (1974) Alpha fetoprotein: An index of progressing of hepatoma, and a target for immunotherapy. *Ann. Surg.*, **18**, 599.

PEOPLE'S REPUBLIC OF CHINA, The Co-ordinating Group for the Research of Liver Cancer (1974) Application of serum alpha feto-protein assay in mass survey of primary carcinoma of liver. *Am. J. Chin. Med.*, **2**, 241.

SILVER, H. K. B., GOLD, P., FEDER, S. & SHUSTER, J. (1973) Rodioimmunoassay for alpha$_1$-fetoprotein. *Proc. Natl Acad. Sci. U.S.A.*, **70**, 526.

SILVER, H. K. B., KARIM, K. A., ARCHIBALD, E. A. & SALINAS, F. A. (1979*a*) Serum sialic acid and sialyltransferase as monitors of tumour burden in malignant melanoma patients. *Cancer Res.*, **39**, 5036.

SILVER, H. K. B., KARIM, K. A. & SALINAS, F. A. (1979) Identification of malignant melanoma tumor-associated serum sialylglycoprotein independent of acute phase reactants. *Proc. Am. Assoc. Cancer Res.*, **20**, 50.

SILVER, H. K. B., RANGEL, D. M. & MORTON, D. L. (1978) Serum sialic acid elevations in malignant melanoma patients. *Cancer*, **41**, 1497.

TE VELDE, E. R., BERRENS, L., ZEGERS, B. J. M. & BALLIEUX, R. E. (1979) Acute phase reactants and complement components as indicators of recurrence in human cervical cancer. *Eur. J. Cancer*, **15**, 893.

VAN BEEK, W. P., SMETS, L. A. & EMMELOT, P. (1973) Increased sialic acid density in surface glycoprotein of transformed and malignant cells—a general phenomenon? *Cancer Res.*, **33**, 2913.

WARD, M. A., COOPER, E. H. & HOUGHTON, A. L. (1977*a*) Acute phase reactant proteins in prostatic cancer. *Br. J. Urol.*, **49**, 411.

WARD, M. A., COOPER, E. H., TURNER, R., ANDERSON, J. A. & NEVILLE, A. M. (1977*b*) Acute-phase reactant protein profiles: An aid to monitoring large bowel cancer by CEA and serum enzymes. *Br. J. Cancer*, **35**, 170.

WARREN, L. (1959) The thiobarbituric acid assay of sialic acid. *J. Biol. Chem.*, **234**, 1941.

WEISS, J. F., MORANTZ, R. A., BRADLEY, W. P. & CHRETIEN, P. B. (1979) Serum acute-phase proteins and immunoglobulins in patients with gliomas. *Cancer Res.*, **39**, 542.